# A Survey of Italian Dairy Farmers’ Propensity for Precision Livestock Farming Tools

**DOI:** 10.3390/ani9050202

**Published:** 2019-04-28

**Authors:** Fabio Abeni, Francesca Petrera, Andrea Galli

**Affiliations:** CREA Research Center for Animal production and Aquaculture, via A. Lombardo, 11 - 26100 Lodi, Italy; francesca.petrera@crea.gov.it (F.P.); andrea.galli@crea.gov.it (A.G.)

**Keywords:** dairy cow, estrus detection, precision livestock farming, survey, welfare monitoring

## Abstract

**Simple Summary:**

The use of information and technology in precision livestock farming (PLF) to improve animal health, welfare, and farm efficiency is a developing area with great scientific and commercial interest. To gain a better understanding of the use of PLF tools by Italian dairy farmers and to analyze their perceptions of the importance of new technologies, a survey was carried out in the province of Cremona, an area of well-recognized economic importance for the Italian dairy industry. Technicians at the Provincial Breeder Association interviewed 490 farmers (who represent 82% of the associated dairy farms in this province) to collect information on the parameters currently measured by PLF tools on their dairy farms. The survey provided evidence of the interest in, and significant presence of, PLF tools for the individual monitoring of dairy cows. Most farmers reported using sensors for automated milk yield recording and estrus detection. Although the use of sensors for the detection of mastitis and estrus did not appear to be widespread, this technology was considered very important for the improvement of herd management. Propensity for PLF technology use was greater in farmers managing larger herd sizes. The benefit-to-cost ratio was evaluated as the most important factor in determining whether to use a particular tool. In addition, the farmers stated that before purchase, they considered the time required to manage the data generated by technology as an important issue.

**Abstract:**

A targeted survey was designed with the aim of describing the diffusion of precision livestock farming (PLF) tools in one of the most intensive dairy farming provinces in Italy. Technicians at the Provincial Breeder Association of Cremona interviewed 490 dairy farmers and obtained data regarding the role and age of the respondents; the land owned by the farmers; their herd sizes (HS, lactating plus dry cows; small HS < 101, medium HS 101–200, large HS > 200 cows/herd); their average 305 day milk yield (low MY < 9501, medium MY 9501–10,500, high MY > 10,500 kg/head); the cow to employed worker ratio (low CW < 33, medium CW 33–47, high CW > 47 cows/worker); the use of PLF tools to monitor production, reproduction, and health; and the criteria and motivations for investing in PLF tools. The use of automated MY recording and estrus detection systems was primarily associated with HS (more present in larger farms), followed by MY (more present in more productive farms), and then CW (more present with a high cow: worker ratio). Concern about the time required to manage data was the most common subjective issue identified as negatively affecting the purchase of these tools. The future of PLF use in this region will depend upon the availability of an effective selection of tools on the market.

## 1. Introduction

The definition of precision livestock farming (PLF) is the “management of livestock by continuous automated real-time monitoring of production/reproduction, health and welfare of livestock, and environmental impact”. [1]. The use of PLF tools by farmers can play an important role in the development of efficient production of healthy livestock [2]. The commercial availability of the first automatic milking system (AMS) in 1992 is generally considered as an important step toward the introduction of new digital technologies in dairy farms [3]. Accordingly, first generation sensors, such as those for automated milk yield recording (AMYR), milk color evaluation, and conductivity measurement, were introduced with the AMS [4]. At the same time, AMS is an example of innovation that implies changes in herd management [5] with possible changes in milk yield (MY) and quality [6,7,8] that farmers must consider before implementation.

The available tools include systems for AMYR and milk quality monitoring, automated estrus detection (AED), calving time forecast, detection of health problems (namely lameness and mastitis), and feeding-related metabolic problems. More specific applications of the current tools would potentially help in decision-making processes by aiding in the early detection of health or well-being problems in individual cows and the application of targeted corrective practices [9], for example, the early detection of decreased rumen activity at the beginning of a heat wave [10]. According to several papers, activity monitoring tools can correctly predict ovulation and suggest the right time for artificial insemination [11], as they have been found to detect 70% of cows considered to be in estrus [12]. These tools can also give estrus-related information (i.e., duration, intensity), which could be useful in deciding the timing of insemination [13].

Steeneveld et al. [14] reported that having estrus detection sensor systems does not improve reproduction performance per se. Instead, they suggested that the potential technological improvement expected by the use of the sensors did not automatically materialize just through their use, probably because farms needed more information and guidance on how to use the output from the sensor systems [15].

Economic criteria to support the use of PLF tools are crucial and include a change in the capital to labor ratio and the technical efficiency [3], the net present value, the benefit-to-cost ratio, the internal rate of return, and the discounted payback period, as reported by Rutten et al. [16].

In their survey, Borchers and Bewley [17] reported that US dairy farmers give priority to the introduction of technologies for AMYR, followed by cow activity monitoring systems for AED, and systems for automated mastitis detection (AMD). Several studies [18,19] focused on determining the definition of applicative software tools to evaluate the convenience threshold for investing in AED technology. Bewley et al. [20] reported that lack of familiarity with the available technologies (55%) together with a high cost-to-benefit ratio (42%) limit the use of innovative technology, while 7% of surveyed farmers considered compatibility with other technologies as a problem. Contrary to the wide range of tools available to support farm animal well-being [9], their adoption by dairy farmers is today still limited [17].

In Italy, the Province of Cremona is an important production area of cow’s milk and cheeses. In 2016, this region produced as much as 1,216,579 tons of milk, 10.5% of the milk delivered nationally [21], which was mainly used for the production of protected designation of origin products, predominantly Grana Padano cheese.

As little was previously known about the propensity of Italian farmers to use new technologies, the aims of this article were (1) to collect information on the parameters currently measured in Cremona dairy farms to gain a better understanding of PLF tool diffusion in this area; (2) to investigate which parameters are considered the most interesting by farmers to support health management and improve efficiency on their farms; and (3) to understand the most important considerations of farmers when purchasing new technologies.

## 2. Materials and Methods

### 2.1. Farm Selection and Data Collection

This study was carried out as a collaboration between the CREA and the Provincial Breeder Association (APA) of Cremona. The APA technicians surveyed 490 dairy farmers (representing 82% of the dairy farms in the province of Cremona [22]) during the summer and autumn of 2016. The survey was planned according to recent survey design literature in this field [17,23] in order to attain comparable results to other studies on this topic. The farmers’ identities were unknown to the researchers.

The questionnaire consisted of three sections with a total of 56 questions, 15 open-ended and 41 multiple-choice. The open-ended questions were designed to collect quantitative data about the farms and the specifications (commercial brands included) of PLF tools not included in the submitted, pre-compiled list. The first section collected the following general information: farm locality and area within the province of Cremona (cremasco, northern; cremonese, central; casalasco, southern); the land owned by each farmer (ha); the current herd size (HS, lactating plus dry cows) and HS variation compared to 2015 (as %); the mean 305 day milk yield (MY) per cow (MY, kg/305 days); the total number of employed workers (EW) for farmland and herd management; whether a conventional or automatic milking system was used (CMS or AMS); the age of the respondents (<31, 31–40, 41–50, 51–60, and >60 years); and the on-farm role of the interviewed person (owner or family member; president or vice president; manager, supervisor, or herdsman; employed worker; other).

In the second section of the questionnaire, the farmers were asked to choose one of three options for a variety of tools: (a) “in use”—the tool was present and being used on the farm; (b) “int”—the tool was not present, but the farmer was interested in buying it; and (c) “not int”—the tool was not present and the farmer was not interested in buying it. To avoid misunderstandings about the tool classification, commercial brands were recorded. The technologies used to automatically measure the physiological, behavioral, and production parameters of individual animals were divided into three categories: sensors for automated estrus and health monitoring (or well-being status); sensors for automated feeding behavior and metabolic problem monitoring; and sensors for MY recording and automated mastitis detection.

In the third section, using a Likert scale of 1 (of little importance) to 5 (of great importance), the farmers were asked to rate [24] the importance of criteria used to evaluate the importance of PLF tools. The listed criteria were as follows: (1) the benefit-to-cost ratio; (2) the total investment cost; (3) the user-friendly degree of the new tool; (4) third party (other farmers) opinions on tool performance; (5) the availability of good local technical assistance; (6) the possibility of linking a tool with software from existing and new systems for data integration; and (7) time required by the new technology for information and data management. These criteria are similar to those listed by Borchers and Bewley [17]. Finally, we asked the farmers to rate the extent to which the following factors would encourage them to buy new technologies: (1) reduction of labor costs or improvement of labor efficiency; (2) improvement of estrus and health monitoring; and (3) increased farm profitability.

### 2.2. Statistical Analyses

Statistical analyses were conducted on all 490 surveys using SAS Version 9.4 (SAS Institute Inc., Cary, NC, USA) and R Version 3.1.1 (R Foundation for Statistical Computing, Vienna, Austria). Starting from a population of 600 farms in the region and considering a proportion of 0.5 vs. 0.5 (the most protective hypothesis) in order to evaluate possible differences with a bound on the estimation error of 0.05, the required sample was 240 [25]. However, we decided to record a higher number of farms, because the technicians had easy and quick access to the farms without extra costs.

The mean, median, standard deviation, and first and third quartile of general data were calculated using the MEANS procedure (SAS Institute Inc., Cary, NC, USA).

Least-squares means were calculated using the General Linear Model (GLM) procedure for all data or divided into the following categories: HS (small HS (<101 cows/herd), *n* = 129; medium HS (101–200), *n* = 197; and large HS (>200), *n* = 164), herd average 305 day milk yield (low MY (<9501 kg/head), *n* = 172; medium MY (9501–10,500), *n* = 182; and high MY (>10,500), *n* = 137); and the cow to unit (CW) ratio (low CW (<33 cows/EW), *n* = 146; medium CW (33–47), *n* = 151; and high CW (>47), *n* = 175). The adopted stratifications were planned to attain the most balanced sampling across the three different homogeneous agricultural areas of the province of Cremona in order to avoid possible bias in the evaluation of the main tested effects.

Chi-squared analyses were performed using the FREQ procedure in SAS Version 9.4 (SAS Institute Inc., Cary, NC, USA) to compare the differences among the HS, MY, and CW categories across the measured parameters.

To identify the main structural (general information data on the farm and respondent’s age) and subjective (score assigned to criteria and motivations) factors affecting the probability of using at least one tool for AED, AMYR, or AMD, six logistic regression analyses were performed. Through this, considering the statistical significance of each factor within a regression, the relative odds ratios (OR) were highlighted for further discussion.

The initial model always included the farmers’ ages (class, 1 to 5) together with all of the structural variables (models 1 to 3, one for each family of tools) or with all of the subjective evaluation criteria (models 4 to 6, one for each family of tools). To evaluate the possible effects of multicollinearity, the variance inflation factors were calculated using the “vif” function from the “car” package [26], and were found to always be < 10. A backward stepwise selection was performed until the retained variables in the model had a Wald statistic with *p* < 0.15.

To show the distribution of the classified answers to the continuous explanatory structural variables in logistic regressions, frequencies were plotted as spinograms, as described by Everitt and Hothorn [27]. These plots were derived from stacked bar plots. As described by Radtke et al. [28], the metric variable on the x-axis was broken into intervals to create stacked bar plots, where the width of each bar was proportional to the relative frequency of the x-value and the height was proportional to the conditional frequency of the y-category.

## 3. Results

### 3.1. General Information about the Surveyed Farms

Approximately 93% of the respondents were the owners of the farm or family members. The most represented age class was 41–50 years (34% of the sample), followed by 51–60 years (30%), 31–40 years (15%), >60 years (11%), and only 8% of the farmers were less than 31 years of age.

The descriptive statistics of the 490 dairy farms involved in the survey are shown in Table 1. Both the mean and median of each variable are reported to provide a better description of not normally distributed data (namely land owned by farmers and herd size). The area of owned farmland and the HS varied largely among farms (coefficient of variation: 93% and 73%, respectively). As a consequence, the total EW and the cow to EW ratio revealed high variability, and MY was the only parameter to display a lower deviation.

### 3.2. Information on the Parameters Measured on the Dairy Farms

Table 2 shows the percentages of farmers that use PLF technologies for automated monitoring of estrus and animal well-being (a), feeding behavior and metabolic problems (b), and individual MY and mastitis detection (c). The percentages of farmers not using the devices and their interest in them (or not) are also shown. Only 3.4% of the farmers stated that they have an automated milking system; therefore, it made no sense to compare this small group with the remaining farms for the other PLF tools due to an expected bias.

The most commonly used technologies to detect estrus were identified as pedometers (34.5% of the total respondent population) and activity meters (29.0%) measuring leg or neck activity, whereas the least used parameter in this category was milk progesterone (used by one farm only).

Approximately half of the respondents stated that they are interested in monitoring locomotion problems or milk progesterone. About 58% of respondents were not interested in using PLF tools to monitor calving moments, position, and location, or cow physiological parameters (body temperature, respiration rate, or heart rate). Automated systems for monitoring hoof health or locomotion problems were found to be rarely used (one farm only) but were of interest for 50% of the respondents.

A combined system to measure cow activity plus rumination was present in 71 farms, and 58.4% of respondents had no such system but expressed an interest in it. Overall, 48.6% of farms used at least one sensor for AED (considering tools for measuring leg or neck activity, progesterone in milk, and neck activity plus rumination), and 43.4% of respondents were interested in this technology. Other technologies for the automated monitoring of feeding behavior or for metabolic problem detection were rarely used (0.0–1.0%), and over 66% of respondents were not interested in measuring rumen pH, rumen temperature, cow body condition score (BCS), or body weight (BW). Over 63% of them were interested in measuring milk beta-hydroxybutyrate (BHB), while over 55% were not interested in measuring milk urea. Methane emissions were considered the least interesting parameter.

The farmers’ responses indicated that AMYR was the most frequently measured parameter by PLF technologies (39.4%), and only 9.2% of farmers were not interested in it. Milk electrical conductivity (EC) was the most measured AMD parameter (23.3%), and 44.1% of farmers were not using it, but expressed interest. Over 81% of the respondents expressed interest in measuring the milk somatic cell count (SCC), and over half of them were interested in measuring milk components, whereas milk lactate dehydrogenase (LDH), milk color, and temperature were not of interest for over 55% of those interviewed. A total of 23.6% of the farmers used at least one AMD sensor, but more than 63.1% were interested in measuring one or more of the listed parameters. Automated systems for monitoring milk composition were poorly represented, but over 51% of the respondents were not using them but were interested in their use.

### 3.3. Distribution and Interest in Technologies Based on Categories

The percentage of respondents using at least one sensor for AED was greater in farms with larger herds than in those with smaller herds (73.8% vs. 20.9%) as well as in farms with higher productivity compared to those with low productivity (64.2% vs. 32.6%), and in those with higher cow-to-worker ratios compared to those with lower ratios (61.7% vs. 34.2%). The greatest difference was observed for the use of pedometers—61.6% of the farms with >201 cows and only 10.1% of farms with <101 cows.

Automatic MY recording systems were the most used tools in our survey, and were present in the milking systems of 67.1% of larger herds vs. 13.2% of smaller herds; in 48.9% of high productivity vs. 26.7% of low productivity herds; and in 53.7% of the high cow to worker ratio vs. 22.0% of the low cow to worker ratio farms. Sensors for AMD were present in 44.5% of farms with large herd sizes, in 31.4% of those with high productivity levels, and in 34.3% of farms with high cow-to-worker ratios.

### 3.4. Prediction of the Presence of a Tool in the Farm Based on General Information

Table 3 shows the output of the three logistic regressions that link general farm information to the presence of at least one tool for AED, AMYR, or AMD. The AED presence was strongly associated with the herd size, milk yield, and land owned by farmers; the age of the farmer was retained in the model because its *p*-value was below 0.15. The presence of tools for AMYR was significantly linked with the herd size and land owned by farmers, but the milk yield was also retained in the model (*p*-value < 0.15). The only independent variables associated with the presence of AMD systems were the herd size and the age of the farmer.

### 3.5. Criteria Considered in Evaluating Possible Investment in PLF

With regard to the criteria and motivations considered important by producers from the aspect of potential investment in PLF tools, farmers assigned higher scores (mean ± SD) to the benefit-to-cost ratio, the availability of good local technical assistance, and the daily time required by the new technology for information and data management (4.74 ± 0.64, 4.67 ± 0.63, and 4.50 ± 0.80, respectively); in contrast, third party opinions on tool performance were not rated as important (3.45 ± 0.63).

Of the proposed motivations for farmers to purchase a PLF tool, increased farm profitability (4.98 ± 0.26), reduction of labor costs or improvement of labor efficiency (4.90 ± 0.43), and improvement of estrus detection and health monitoring (4.84 ± 0.53), were considered of great importance (score: 5) by 98.6%, 93.5%, and 88.6% of the respondents, respectively.

### 3.6. Prediction of the Presence of a Tool in the Farm Based on Criteria and Motivation Scores

Table 4 shows the output of the three logistic regressions that linked the score assigned by farmers to the criteria and motivation considered when evaluating possible investment in PLF to the presence of at least one tool for AED, AMYR, or AMD. These factors had significant different associations with the presence of each of the three categories of tools.

The use of AED tools was positively affected by the user-friendly degree of a new tool as well as the benefit-to-cost ratio, but it was negatively affected by concern about the time required to acquire the necessary skills for tool management and by the expectation of being able to detect early health problems to prevent pathologies. The presence of AMYR tools was positively related to the judgement of a third party on the effectiveness of this tool, and negatively related to concern about the time required to acquire skills. The presence of AMD systems was positively related to the benefit-to-cost ratio evaluation and third-party judgement but negatively related to the evaluation of the total cost of the investment and concerns about time required to acquire skills.

**Figure 1:**
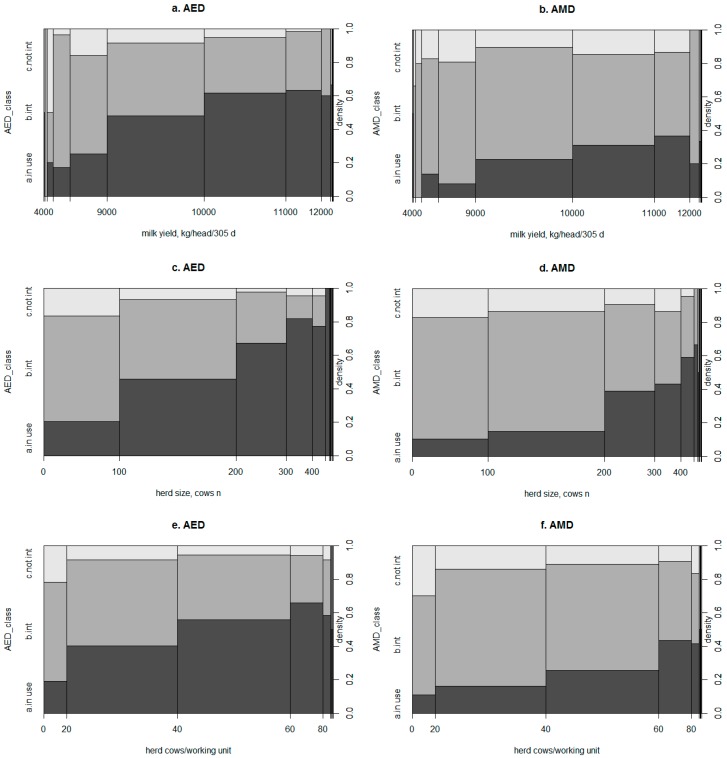
Spinograms for the distribution of farms with at least one sensor for (**a**,**c**,**e**) automated estrus detection (AED) or for (**b**,**d**,**f**) automated mastitis detection (AMD) with the (**a**,**b**) herd average 305- day MY (kg/head), (**c**,**d**) herd size (lactating plus dry cows n) and (**e**,**f**) cows per working unit ratio (n) as independent variables. The three different areas represent the proportions of interviewed farmers that used (dark-gray), were interested (medium-gray), or were not interested in using (light-gray) at least one form of the technology. The widths of the bars correspond to the relative frequencies of x, and the heights correspond to the conditional relative frequencies of y in every x interval.

Alongside the results from the first logistic regression analysis, Figure 1 shows a similar pattern for the AED and AMD systems regarding the HS, MY, and CW categories. The presence of both tools increased mostly in farms with >200 cows. Specifically, the presence of an AMD system was more than double in farms with 200–300 cows compared to those with 100–200 cows. The lack of interest in these two kinds of systems was concentrated in the lower range of CW (0 to 20). However, for both systems, interest was greater in larger herd sizes.

## 4. Discussion

The survey results indicated that daily MY, mastitis, and cow activity are the most commonly measured parameters by technology. This is in accordance with a survey conducted by Borchers and Bewley [17] in the USA. The individual AMYR system was considered to be the most useful PLF tool. It is available on all AMS; however, only 3.4% of farmers surveyed used AMS alone or with CMS. As shown in Table 2, it was clear that within each area of “purpose-oriented” tools (estrus and well-being monitoring; feeding behavior and metabolism; milk yield, quality, and mastitis detection), no more than two kinds of tools were predominant. This may be attributable to the availability of affordable products together with the reliability of technologies for the target purposes over the recent decade.

Herd size was the leading factor in determining the presence of AED, AMYR, and AMD systems, as evidenced in Table 3. According to Gargiulo et al. [29], a big herd may benefit more than a small one from the introduction of automated systems to monitor individual cows, reflecting the farmers’ attempts to address labor issues and the need to be consistent in protocols to monitor and manage larger numbers of cows. The area of land owned by farmers and the average MY of the herd were positively related to the presence of AED and AMYR systems but were not related to AMD. This could be attributable to a relative prudence toward kinds of products that do not perform (on average) as well as AED in terms of sensitivity and specificity, as reviewed by Rutten et al. [30]. Concern about the time required to manage data and information was negatively related to the presence of all three categories of tools. This suggests the important role of having user-friendly and time-saving interfaces for the digital control of tools [31]. Regarding the presence of AMYR sensors for precision dairy farming, their helpfulness is associated with the importance of knowing daily individual MY values and their variation to aid in feeding management (grouping or individual feed supplementation) and to support breeding and culling decisions. Moreover, this parameter, if used together with other parameters, such as cow activity, rumination data, or milk EC, could support farmers in monitoring reproductive events or in the early detection of cows with health problems. As an example, using a logistic regression, Van Hertem et al. [32] reported the significance of daily MY data to detect lameness in cows. However, it is not generally possible to automatically merge data from two or more separated systems (commercialized by different brands), unless it is allowed by a specific commercial contract.

The presence of AMYR sensors was five times higher in farms with >200 cows compared to those with <101 cows (67.1% vs. 13.2% in large HS and small HS), but the difference was only 1.8 times higher for high MY compared to low MY farms and 2.4 times for high CW compared to low CW farms. These results were confirmed by the first logistic regression analysis, where the most significant independent variable to determine the presence of AMYR technology was found to be HS. Indeed, because milking systems with AMYR sensors require considerable investment, they are more likely to be used in larger herds than in smaller ones (as many cows need to be monitored simultaneously).

In general, automated activity monitoring tools are the most commonly used systems for the detection of estrus in cows. In a study of Wisconsin farms, Bewley et al. [33] identified HS as a significant factor in determining the labor efficiency, expressed as the cow-to-worker ratio. In our study, the logistic regression showed that HS was a significant factor in determining the presence of all three kinds of PLF technology, but CW was not. The main reason for this apparent discrepancy could be attributable to the different farm labor organization in the Province of Cremona and to the great difference in hectares of land owned by farmers and consequently, in employed workers for farmland management.

Steeneveld and Hogeveen [34] characterized Dutch farms with and without sensor systems. In their survey, the only difference found among CMS farms without sensors, CMS farms with sensors, and farms using AMS was the HS. Indeed, the HS was significantly higher in CMS farms with sensors compared to the other two, and the number of labor hours per cow per week was lower in CMS farms with sensor systems than in those without sensors.

Some questions have arisen considering the farmer’s expectations and the actual knowledge (from the first European surveys) of the real performances and advantages of using some PLF tools. As presented in a recent review by Hostiu et al. [35], factors other than economics can motivate dairy farmers to adopt new technologies. If possible, labor reduction represents a goal for the farmer, as it can lead to a generally improved quality of life due to a less stressful management system [35]. Possible labor reduction was considered to be an important motivation factor by farmers for investing in sensor systems in a study by Steeneveld et al. [14], but no information about this aspect was reported by Lawson et al. [36], nor by Borchers and Bewley [17]. Further, the investment in AMS is a typical case where the technology increases the capital costs for milk without a clear substitution of capital for labor [3].

The most important factor for considering a PLF investment was found to be the benefit-to-cost ratio, as also shown by Borchers and Bewley [17]. This preference indicates the importance of actions by universities, companies, and other services to support farmers in their economic analysis. Unlike US producers [17], who were found to consider the total investment cost the second most important criterion for evaluating possible investment in PLF, our farmers gave it a low score. A direct explanation for this difference is not easy because, to our knowledge, there has been no US–Italy comparative study on dairy farmers’ propensity for investments. Furthermore, considering the very similar choices available in our questionnaire compared to Borchers and Bewley [17], the different rankings in the answers on financial matters may have been influenced by the presence of the interviewer.

The use of all three kinds of tools was related to both third-party opinion and time required for data management. In contrast, only the AED and AMD tools were related to both the farmer’s age and the benefit-to-cost ratio; this is probably because of their usefulness and the immediate economic benefits when compared to sensors for AMYR.

As expected, farmers using at least one system evaluated the possible investment from a more operative point of view. Even though a different statistical approach was used, our study confirmed the discrepancy found by Borchers and Bewley [17] for the importance assigned to the listed criteria between farmers using or not using PLF tools. In their survey [17], this was evident for the weights attributed to the availability of local support and the time involved in using the technologies. In our case, this was evident for the third party opinions on the tool’s performance (for AMYR and AMD), the possibility of linking software from existing and new systems for data integration (for AED), and the time required by the new technology for information and data management (for AED and AMYR).

Regarding the AED, Neves and LeBlanc [23] conducted a survey in Canada and divided the answers on the considered criteria between farmers not using and using AED. The majority of Canadian respondents without AED (53%; 148/279) answered that they were satisfied with their current herd reproductive performance, and approximately 39 of the respondents reported a perceived lack of economic value from AED (i.e., the costs were too high relative to the benefits). The most frequently reported reason for the investment by owners of AED systems was the desire to improve their herd’s reproductive performance (81%; 180/220). Respondents also indicated that consultation with other farmers had been the most important factor influencing their decision to adopt the technology.

The majority of respondents in our survey replied that third party opinion on tool performance was the least important factor when considering investment in PLF; however, while it did not affect the presence of AEDs, it significantly affected the use of AMYR and AMD systems on the farms.

In their survey on farmers’ preferences for automated lameness detection systems, Van De Gucht et al. [37] demonstrated the positive effect of providing correct information to farmers about the possibility of preventing disease in determining their opinions regarding the choice to invest in the system. In our survey, the farmers were asked for their preferences and criteria without previous supplementary information, because our aim was to assess the farmers’ attitude for PLF investments, based on their actual knowledge of the most common PLF technologies and the new measured parameters.

## 5. Conclusions

This survey represents a first attempt to describe the adoption of PLF technologies in one of the most intensive and specialized regions for dairy farming and cheese production in Italy. We attempted to relate the use of PLF technology in this area to several structural features of the farms in addition to the investment evaluation criteria of the farmers. The tools for automated milk recording represented the most used PLF technology, followed by systems for automated estrus detection. There was strong evidence that herd size (i.e., the productive power of the farm) is a driving variable for this kind of investment. There were differences among the driving criteria for the farmers’ decisions of whether to purchase a tool or not. However, the financial aspect did not seem to be the leading factor in the evaluation by the farmers. Many of the next steps in PLF development and promotion will depend upon the provision of an astute and efficient selection of tools on the market.

## Figures and Tables

**Table 1:** Descriptive statistics of the 490 dairy farms involved in the survey.

Item	Mean	Standard Deviation	Median	1^st^ Quartile	3^rd^ Quartile
Land owned by farmers, ha	96.2	89.7	70.0	42.0	115.0
Herd size (dry + lactating cows), n	188.7	137.0	154	98	239
Herd size variation from the previous year, %	1.10	12.22	2.21	−2.89	6.57
Herd average 305-day milk yield, kg/cow	9827.3	1346.5	9870	9121	10,599
Total employed workers, n	4.3	2.2	4.0	3.0	5.0
Cows per working unit, n	43.3	19.1	42.3	30.6	53.8

**Table 2:** Percentages of respondents using, interested, or not interested in using precision livestock farming (PLF) sensors to measure the listed parameters for the automated monitoring of estrus and animal well-being (a), feeding behavior and metabolic problems (b), and individual MY, milk quality, and mastitis detection (c) in the sample farms.

Parameter	Using	Interested in	Not interested in	No Answer
a. Technologies for automatic estrus detection and animal well-being monitoring				
Leg activity (pedometer)	34.5 (169) *	37.6 (184)	22.7 (111)	5.3 (26)
Neck activity (activity meter)	29.0 (142)	41.4 (203)	25.5 (125)	4.1 (20)
Hoof health/locomotion problems	0.2 (1)	50.2 (246)	49.2 (241)	0.4 (2)
Milk progesterone	0.2 (1)	47.8 (234)	51.2 (251)	0.8 (4)
Calving alert	1.0 (5)	31.4 (154)	58.8 (288)	8.8 (43)
Animal position	3.3 (16)	25.7 (126)	68.4 (335)	2.7 (13)
Animal location	0.4 (2)	13.1 (64)	85.1 (417)	1.4 (7)
Body temperature, heart, and breathing rate	0.4 (2)	22.5 (110)	76.5 (375)	0.6 (3)
b. Technologies for automatic monitoring of feeding behavior and metabolic problems				
Rumination and cow activity	14.5 (71)	58.4 (286)	23.3 (114)	3.9 (19)
Chewing activity	0.4 (2)	41.6 (204)	57.6 (282)	0.4 (2)
Rumen pH	0.2 (1)	32.9 (161)	66.5 (326)	0.4 (2)
Body condition score (BCS)	0.2 (1)	31 (152)	68.6 (336)	0.2 (1)
Body weight (BW)	1.0 (5)	26.9 (132)	71.8 (352)	0.2 (1)
Rumen temperature	0.2 (1)	24.9 (122)	74.9 (367)	0.0 (0)
Milk beta-hydroxybutyrate (BHB)	0.6 (3)	63.3 (310)	34.7 (170)	1.4 (7)
Milk urea	0.4 (2)	44.1 (216)	54.7 (268)	0.8 (4)
Methane emissions	0.0 (0)	10.4 (51)	89.6 (439)	0.0 (0)
c. Technologies for automatic MY, milk quality, and mastitis detection				
Daily milk yield	39.4 (193)	46.7 (229)	9.2 (45)	4.7 (23)
Milk somatic cell count (SCC)	1.0 (5)	81.6 (400)	16.5 (81)	0.8 (4)
Milk electrical conductivity (EC)	23.3 (114)	44.1 (216)	32.0 (157)	0.6 (3)
Milk components (e.g., fat, protein, SCC)	1.8 (9)	51.8 (254)	45.1 (221)	1.2 (6)
Milk color	3.3 (16)	40.0 (196)	55.7 (273)	1.0 (5)
Milk lactate dehydrogenase (LDH)	0.2 (3)	41.0 (201)	58.8 (288)	0.2 (1)
Milk temperature	1.6 (8)	36.7 (180)	60.8 (298)	0.8 (4)

* Frequency in parenthesis.

**Table 3:** Logistic regression output and obtained odds ratios for the prediction of the presence of a tool for automated estrus detection (AED), milk yield recording (AMYR), and mastitis detection (AMD) in the farms, as determined by the analysis of general farm information.

Variable	*β* Estimate	SE	*p*-Value	Odds Ratio	95% CI
Automated estrus detection (AED)					
(Intercept)	−3.48800	0.88260	<0.001	0.0306	0.0052–0.1659
Farmer’s age (class 1 to 5)	−0.15480	0.08805	0.079	0.8566	0.7198–1.0173
Own farmland (ha)	0.00539	0.00218	0.013	1.0054	1.0013–1.0099
Herd size (no. of dairy cows)	0.00423	0.00135	0.002	1.0042	1.0016–1.0070
Herd average 305 day MY (kg/cow)	0.00027	0.00008	0.001	1.0003	1.0001–1.0004
Automated milk yield recording (AMYR)					
(Intercept)	−3.36500	0.82210	<0.001	0.0346	0.0066–0.1672
Land owned by farmers (ha)	0.00505	0.00204	0.013	1.0051	1.0012–1.0093
Herd size (no. of dairy cows)	0.00526	0.00131	<0.001	1.0053	1.0027–1.0079
Herd average 305 day MY (kg/cow)	0.00015	0.00008	0.081	1.0001	1.0000–1.0003
Automated mastitis detection (AMD)					
(Intercept)	−1.68968	0.36779	<0.001	0.1846	0.0879–0.3729
Farmer’s age (classes 1 to 5)	−0.20208	0.09958	0.042	0.817	0.6718–0.9935
Herd size (no. of dairy cows)	0.00554	0.00089	<0.001	1.0056	1.0039–1.0074

*β* = regression coefficient. SE = Standard Error. CI = Confidence Interval.

**Table 4:** Logistic regression output and obtained odds ratio for the prediction of the presence of a tool for automated estrus detection (AED), milk yield recording (AMYR), and mastitis detection (AMD) in the farms, as determined by the analysis of individual convenience factors.

Variable	*β* Estimate	SE	*p*-Value	Odds Ratio	95% CI
Automated estrus detection (AED)					
(Intercept)	0.31410	0.91245	0.731	1.3690	0.2185–8.3691
Farmer’s age	−0.14623	0.08348	0.080	0.8640	0.7325–1.0167
Benefit-to-cost ratio	0.31144	0.15348	0.042	1.3654	1.0162–1.8596
Third party opinions on tool performance	0.13922	0.08506	0.102	1.1494	0.9737–1.3599
The user-friendly degree of the new tool	0.23100	0.09097	0.011	1.2599	1.0569–1.5115
Time needed for information and data management	−0.23370	0.09760	0.017	0.7916	0.6496–0.9543
Improvement of estrus and health monitoring	−0.37110	0.17316	0.032	0.6900	0.4869–0.9653
Automated milk yield recording (AMYR)					
(Intercept)	−0.30732	0.43749	0.482	0.7354	0.3095–1.7338
Third party opinions on tool performance	0.18046	0.08225	0.028	1.1978	1.0209–1.4102
Time needed for information and data management	−0.17403	0.08270	0.035	0.8403	0.7130–0.9877
Automated mastitis detection (AMD)					
(Intercept)	−3.01592	1.08641	0.006	0.0490	0.0047–0.3454
Farmer’s age	−0.17291	0.09641	0.073	0.8412	0.6960–1.0166
Benefit-to-cost ratio	0.51180	0.22292	0.022	1.6683	1.1081–2.6796
Total investment cost	−0.18976	0.10894	0.082	0.8272	0.6680–1.0255
Third party opinions on tool performance	0.22645	0.10287	0.028	1.2541	1.0277–1.5393
The user-friendly degree of the new tool	0.17373	0.11113	0.118	1.1897	0.9643–1.4936
Time needed for information and data management	−0.18919	0.10619	0.075	0.8276	0.6729–1.0242

*β* = regression coefficient. SE = Standard Error. CI = Confidence Interval.
